# Comparison of the incidences of anastomotic leakage when PDSII or LACLON are used in esophago-gastric conduit handsewn anastomosis after esophagectomy

**DOI:** 10.1038/s41598-020-72619-x

**Published:** 2020-09-24

**Authors:** Yusuke Sato, Satoru Motoyama, Akiyuki Wakita, Yuta Kawakita, Yushi Nagaki, Kaori Terata, Kazuhiro Imai, Yoshihiro Minamiya

**Affiliations:** Department of Thoracic Surgery, Akita University Graduate School of Medicine, Akita, 010-8543 Japan

**Keywords:** Oesophageal cancer, Polymers, Gastroenterology

## Abstract

The incidence of anastomotic leakage after esophagectomy remains around 10%. It was previously reported that PDSII rapidly loses tensile strength at pH 1.0 and pH 8.5. By contrast, LACLON degradation is reportedly insensitive to pH. We therefore compared LACLON with PDSII for esophago-gastric conduit, layer-to-layer, handsewn anastomosis. Between January 2016 and January 2020, 90 patients who received posterior mediastinal gastric conduit reconstruction with layer-to-layer handsewn anastomosis (51 using PDSII and 39 using LACLON) at Akita University Hospital were enrolled. The incidence of anastomotic leakage was significantly lower in the LACLON (2.6%, 1/39 patients) than PDSII group (15.7%, 8/51 patients) (p = 0.0268). Multivariable logistic analysis showed the risk of anastomotic leakage was significantly greater with PDSII than LACLON (odds ratio 11.01; 95% CI 1.326–277.64; p = 0.024). The percentages of time the pH was higher than 8 on the gastric conduit side of the anastomosis were 3.1%, 5.7%, 20.9% and 80.5%, respectively, in the four most recent patients. The present study showed that pH at the anastomosis soon after esophagectomy tends to be alkaline rather than acidic, which raises the possibility that this alkalinity facilitates the deterioration of surgical sutures including PDSII.

## Introduction

Based on data from a nationwide Japanese database, as of 2011–2012 the incidence of anastomotic leakage after esophagectomy for esophageal cancer was around 12%, irrespective of whether the esophagectomy was minimally invasive or open^1^. Another report based on data from 12 European countries and the United States showed that the incidence of anastomotic leakage was 16.5% after minimally invasive esophagectomy^2^. Anastomotic leakage after esophagectomy predisposes the patient to critical conditions, including mediastinitis, pleuritis and acute respiratory distress syndrome (ARDS), and increases the likelihood of reoperation and a longer ICU and hospital stay^1,3^. To reduce the incidence of anastomotic leakage, factors to be considered include the anastomotic method (handsewn or stapler), the type of surgical suture used for handsewn anastomosis, the technique used, time required, and blood flow to the esophagus and gastric conduit.

Absorbable monofilament sutures are widely used for handsewn anastomosis of the mucosal layer of the digestive tract. Among those, PDSII (p-dioxanone, ETHICON, New Brunswick, NJ) reportedly loses about 40% of its tensile strength within 1 week at pH 1.0 and 100% of its tensile strength within 2 weeks. In addition, PDSII loses about 25% of its tensile strength after 2 weeks at pH 8.5. This suggests that care must be taken when PDSII is used to close tissues in contact with acidic or alkaline media^4^. By contrast, LACLON (L-lactide:ε + caprolactone, CROWNJUN, Chiba, Japan) retains 100% of its tensile strength after 2 weeks at pH 1.0 or pH 8.5. In the present study, therefore, we compared the incidences of anastomotic leakage when PDSII or LACLON was used for esophago-gastric conduit layer-to-layer handsewn anastomosis after esophagectomy.

## Results

### Patient characteristics

The clinicopathological characteristics of the patients in the PDSII and LACLON groups are summarized in Table 1. There were no significant differences between the two groups with respect to sex, age at surgery, operative procedure, tumor histological type, tumor location, neoadjuvant therapy, pT, pN or pStage.

**Table 1:** Clinicopathological features of 90 esophageal cancer patients.

Characteristics	PDSII (n = 51) (56.7%)	LACLON (n = 39) (43.3%)	p
**Sex**			0.969
Female	8 (15.7%)	6 (15.4%)	
Male	43 (84.3%)	33 (84.6%)	
Age at surgery	65.8 ± 6.5	65.3 ± 8.8	0.971
	(49–78)	(46–80)	
**Operative procedure**			
Open esophagectomy	18 (35.3%)	13 (33.3%)	0.846
Minimally invasive esophagectomy	33 (64.7%)	26 (66.7%)	
**Histological type**			0.284
Squamous cell carcinoma	48 (94.1%)	34 (87.2%)	
Adenocarcinoma	0	2 (5.1%)	
Others	3 (5.9%)	3 (7.7%)	
**Tumor location**			0.462
Upper	14 (27.5%)	10 (25.6%)	
Middle	20 (39.2%)	14 (35.9%)	
Lower	17 (33.3%)	15 (38.5%)	
**Neoadjuvant therapy**			0.634
Chemoradiotherapy	30 (58.8%)	19 (48.7%)	
Chemotherapy	1 (2.0)	1 (2.6%)	
Without	20 (39.2%)	19 (48.7%)	
**Depth of invasion (pT)**			0.086
T0	12 (23.5%)	4 (10.3%)	
T1	15 (29.4%)	14 (35.9%)	
T2	10 (19.6%)	3 (7.7%)	
T3	13 (25.5%)	18 (46.1%)	
T4	1 (2.0%)	0	
**Lymph node metastasis (pN)**			0.573
N0	32 (62.7%)	21(53.8%)	
N1	13 (25.5%)	11 (28.2%)	
N2	5 (9.8%)	4 (10.3%)	
N3	1 (2.0%)	3 (7.7%)	
**Pathological stage (pStage)**			0.150
0	8 (15.7%)	1 (2.6%)	
IA	9 (17.6%)	9 (23.1%)	
IB	7 (13.7%)	1 (2.6%)	
IIA	7 (13.7%)	10 (25.6%)	
IIB	11 (21.6%)	8 (20.5%)	
IIIA	5 (9.8%)	5 (12.8%)	
IIIB	3 (5.9%)	2 (5.1%)	
IIIC	1 (2.0%)	3 (7.7%)	

### Incidence of anastomotic leakage

**Figure 1:**
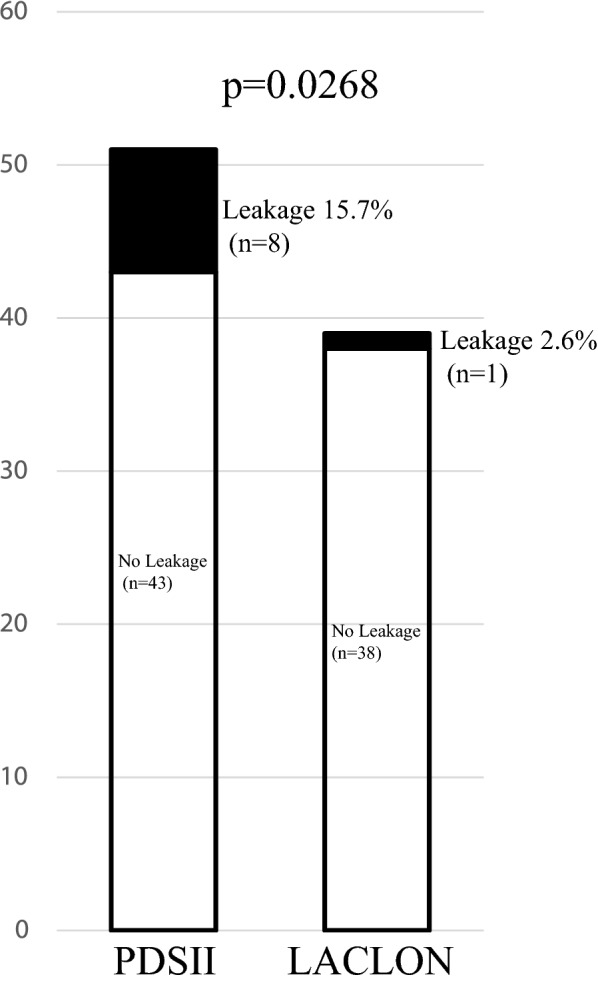
Incidence of anastomotic leakage was significantly lower in the LACLON group (2.6%, 1/39 patients) than the PDSII group (15.7%, 8/51 patients) (p = 0.0268).

The incidence of anastomotic leakage was significantly lower in the LACLON (2.6%, 1/39 patients) than the PDSII group (15.7%, 8/51 patients, p = 0.0268) (Fig. 1). The results of univariable analysis are summarized in Table 2. Surgical suture (PDSII vs. LACLON), sex (female vs. male), neoadjuvant therapy (with vs. without), pN (0 vs 1–3), and pStage (0-IIA vs. IIb-IIIC) were all significant factors affecting the incidence of anastomotic leakage. Moreover, a multivariable logistic analysis taking into consideration sex, age at surgery, neoadjuvant therapy, pT, pN, pStage, and surgical suture showed that the risk of anastomotic leakage was significantly greater in the PDSII than the LACLON group (odds ratio 11.01; 95% CI 1.326–277.64; p = 0.024) (Table 3).

**Table 2:** Univariable analyses of anastomotic leakage.

Variables	Univariate		
	p	Odds Ratio	95% (CI)
**Surgical suture** PDSII (n = 51) vs. LACLON (n = 39)	0.027*	7.070	1.216–134.21
**Age** 65 and older (n = 54) vs. younger (n = 36)	0.664	1.375	0.337–6.876
**Sex** Female (n = 14) vs. Male (n = 76)	0.003*	10.000	2.271–47.58
**Procedure** MIE (n = 59) vs. OE (n = 31)	0.941	1.057	0.258–5.301
**Histological type** Others (n = 8) vs. SCC (n = 82)	0.811	1.321	0.066–8.911
**Tumor location** Upper (n = 24) vs. others (n = 66)	0.052	4.079	0.987–17.98
**Neoadjuvant therapy** With (n = 51) vs. without (n = 39)	0.027*	7.070	1.216–134.21
**pT** 2-4a (n = 45) vs. 0–1 (n = 45)	0.071	3.961	0.893–27.67
**pN** 0 (n = 53) vs. 1–3 (n = 37)	0.037*	6.400	1.100–121.52
**pStage** 0-IIA (n = 52) vs. IIB-IIIC (n = 38)	0.032*	6.727	1.157–127.72

*MIE* minimally invasive esophagectomy, *OE* open esophagectomy, *CI* confidence interval.

*Considered significant.

**Table 3:** Odds ratios of sutures for anastomotic leakage: results of multivariate logistic analyses.

Variables	p	Odds Ratio	95% (CI)
Crude (PDSII vs. LACLON)	0.027*	7.070	1.216–134.21
Adjusted for sex and age	0.016*	10.06	1.449–216.02
Adjusted for sex, age, neoadjuvant therapy, pT, pN and pStage	0.024*	11.01	1.326–277.64

*CI* confidence interval.

*Considered significant.

### pH changes at the anastomosis

**Figure 2:**
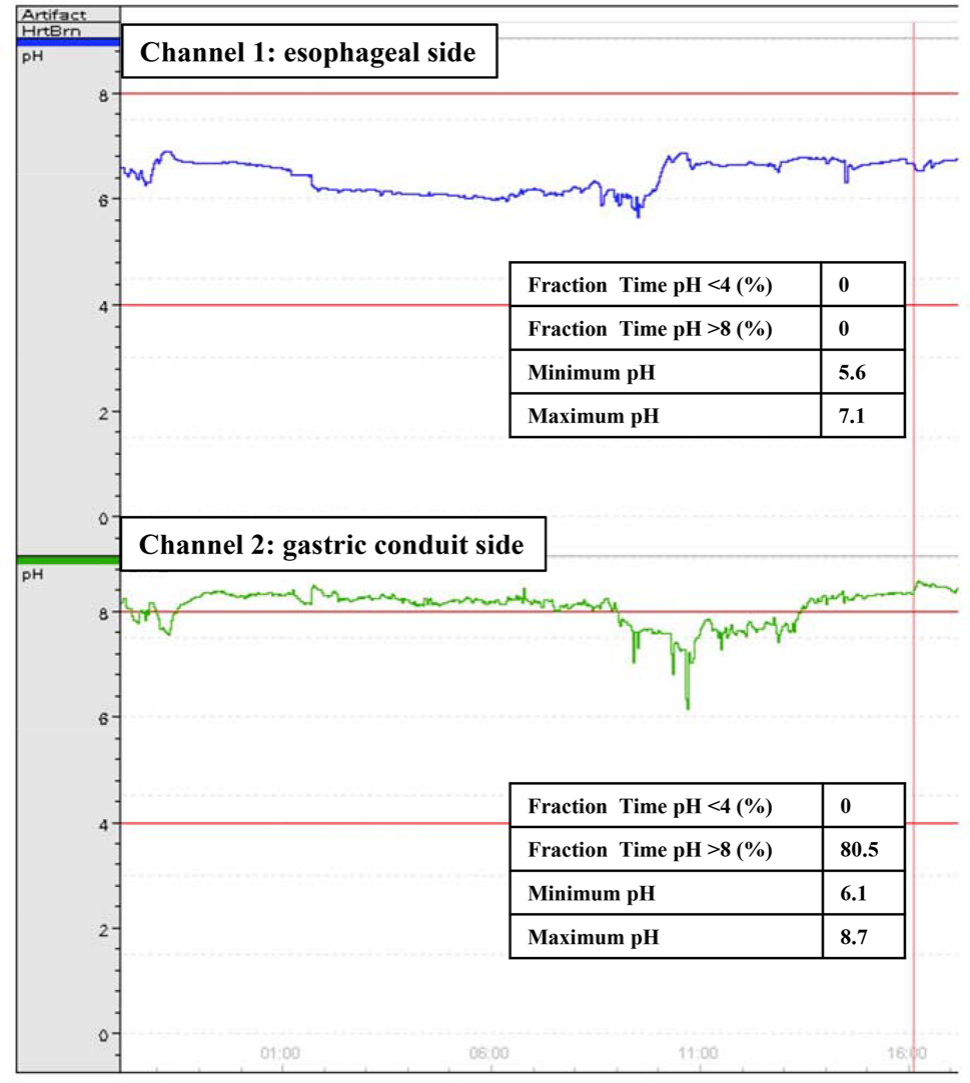
Representative traces recorded from a patient in whom pH was monitored on the esophageal side (channel 1) and gastric conduit side (channel 2) of their anastomosis. Note that in this patient, pH was greater than 8 for 80.5% of the time it was being monitored.

Among the four patients in whom pH was monitored, pH on the esophageal side of the anastomosis was never lower than 4 or higher than 8 (e.g., Fig. 2, channel 1). Similarly, pH was also never lower than 4 on the gastric conduit side of the anastomosis in all cases. However, the percentages of time the pH was higher than 8 on the gastric conduit side were 3.1%, 5.7%, 20.9% and 80.5%, respectively, in the four patients. In Fig. 2, channel 2 shows the changes in pH recorded in the patient exhibiting a pH > 8 on the gastric conduit side of the anastomosis for 80.5% of the time.

### Clavien–Dindo classification of anastomotic leakages

Clavien–Dindo classification of the anastomotic leakages in the two groups are summarized in Table 4. Leakages classified as Grades 2:4, 3a:2, and 3b:2 occurred in the PDSII group, while a Grade 3b:1 leakage occurred in the LACLON group. There were no surgical deaths (Grade 5) in either group.

**Table 4:** Clavien–Dindo classification of anastomotic leakages.

Clavien–Dindo classification	PDSII (n = 8)	LACLON (n = 1)
Grade 1	0	0
Grade 2	4	0
Grade 3a	2	0
Grade 3b	2	1

### Finding of endoscopic examination

**Figure 3:**
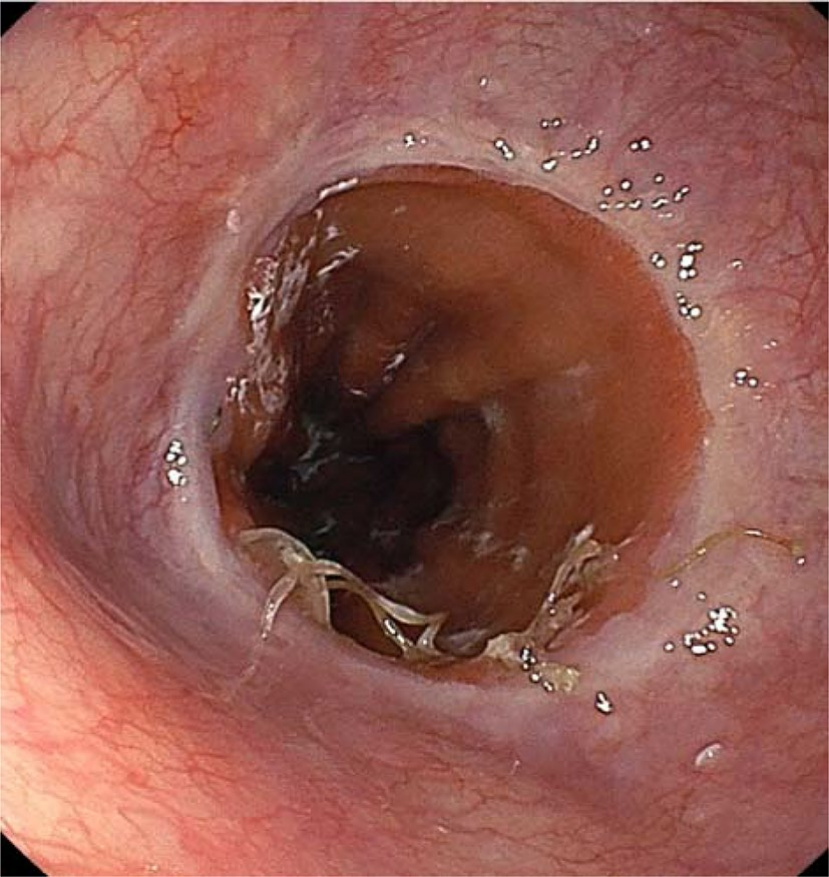
Endoscopic examination of an endo-to-endo, layer-to-layer handsewn anastomosis using LACLON after 3 weeks. The anastomosis is clearly constructed, and some of the LACLON is still in the anastomosis.

Figure 3 shows a representative endoscopic image of a layer-to-layer handsewn anastomosis using LACLON after 3 weeks. The anastomosis was clearly constructed and some of the LACLON is still in the anastomosis.

## Discussion

In this study, we found that the incidence of anastomotic leakage was significantly lower in the LACLON group than the PDSII group. Multivariable logistic analysis showed that the risk of anastomotic leakage was significantly greater in the PDSII group than the LACLON group (odds ratio, 11.01). Moreover, pH monitoring at the anastomosis in the four most recent patients showed that at no time was pH lower than 4 in any of the patients. By contrast, the pH was higher than 8 for some percentage of the time (3.1%, 5.7%, 20.9% and 80.5%) in all four patients. This means the alkalinity may have facilitated the deterioration of the surgical sutures.

It is well known that the denervated stomach retains intraluminal acidity and spontaneously recovers acid production over time after esophagectomy^5–8^. However, those studies monitored pH at the gastric conduit at least one month after the esophagectomy. In the present study, on the other hand, we monitored pH at the oral and anal sides of the anastomosis for 24 h beginning soon after the esophagectomy. We found that pH at the anastomosis soon after the esophagectomy tends to be alkaline rather than acidic. This likely reflects the effect of commonly used perioperative proton pump inhibitor (PPI) administration as well as reflux of bile and pancreatic fluid.

Although Tomihata et al. reported the vulnerability of PDSII to acidic and alkaline environments in 2001^4^, PDSII is still widely used for handsewn anastomosis of the mucosal layer within the digestive tract, including esophago-gastric conduit anastomosis. In their report, those authors warned that care should be taken when PDSII is used to close tissues in contact with acidic or alkaline media. They noted that after 2 weeks of exposure to pH 1.0, PDSII had no tensile strength, while after exposure to pH 8.5, PDSII had lost 25% of its tensile strength. By contrast, LACLON retained 100% of its tensile strength after 2 weeks of exposure to pH 1.0 or pH 8.5. They attributed the rapid degradation of PDSII in acidic or alkaline solution to the presence of a dioxanone component. Most likely the presence of caprolactone, which shows very slow degradation in aqueous media, reduced pH-dependent degradation of the lactide component of the LACLON. The results of the present study are consistent to those reported phenomena.

In a recent analysis of data from 966 patients from 12 European countries and the United States, the leakage rates were determined for five anastomotic techniques, including intrathoracic end-to-side circular-stapled (double stapling and purse string), intrathoracic or cervical side-to-side linear-stapled, and cervical end-to-side handsewn anastomoses after minimally invasive esophagectomy^2^. In that study, the leakage rate for cervical end-to-side handsewn anastomoses was 25.1%, which was significantly higher than the 11.8% for cervical side-to-side linear-stapled anastomoses. Although no information was provided about the type of surgical suture used or the pH changes, it may be that the difference in the incidences of leakage reflects the difference in susceptibility to pH changes between surgical sutures (except LACLON) and linear-stapling. However, limitations of that study noted by the authors are the various biases, including the type of esophagectomy (Ivor Lewis or McKeown procedure), tumor location, surgeon’s individual caseload, surgeon’s career and learning curve. These are general problems associated with multicenter registries and are not unique to that study. From that perspective, the present study has less bias.

There are 2 major imitations in the present study. First, it is not a prospective randomized study. We became aware of LACLON at the end of 2017 and employed it instead of PDSII since beginning of 2018 because we thought it was safer and advantage for the patients. Second, sample size is small. Prospective randomized controlled studies with larger sample size would provide stronger evidences about causal relationship between anastomotic leakage of esophago-gastric conduit handsewn anastomosis and type of surgical sutures. From an ethical viewpoint, however, designing a prospective randomized controlled trial would likely pose a problem, given these clear results.

In summary, the present study showed that pH at the anastomosis soon after the esophagectomy tends to be alkaline rather than acidic, raising the possibility that this alkalinity facilitates the deterioration of surgical sutures. This study also showed the advantage of LACLON over PDSII for esophago-gastric conduit endo-to-endo, layer-to-layer handsewn anastomosis after esophagectomy. We strongly hope these results will make esophageal surgeons aware of the vulnerability of absorbable monofilament sutures to acidic and alkaline environments and contribute to safer esophagectomies worldwide.

## Methods

### Patients

This prospective intervention trial was approved by the Ethics Committee of Akita University School of Medicine (#2306), and all experiments were performed in accordance with the Helsinki Declaration. All study participants provided informed written consent. This clinical trial was registered UMIN-CTR #R000044938 on 06/02/2020. Between January 2016 and January 2020, 164 patients with esophageal cancer received esophagectomy at Akita University Hospital. Among them, 90 patients who received our standard procedure, posterior mediastinal gastric conduit reconstruction with endo-to-endo, layer-to-layer handsewn anastomosis via a left neck wound were enrolled (Table 1). Running suture of the mucosal layer was performed with PDSII in 51 patients (2016–2017), and LACLON in 39 patients (2018–2020). Clinical tumor stages and the treatment strategies were decided by a board composed of radiologists, oncologists, gastroenterologists and surgeons. Pathological stages were determined according to the UICC International Union Against Cancer tumor-node-metastasis (TNM) Classification of Malignant Tumors (7th edition).

### Handsewn Anastomosis

**Figure 4:**
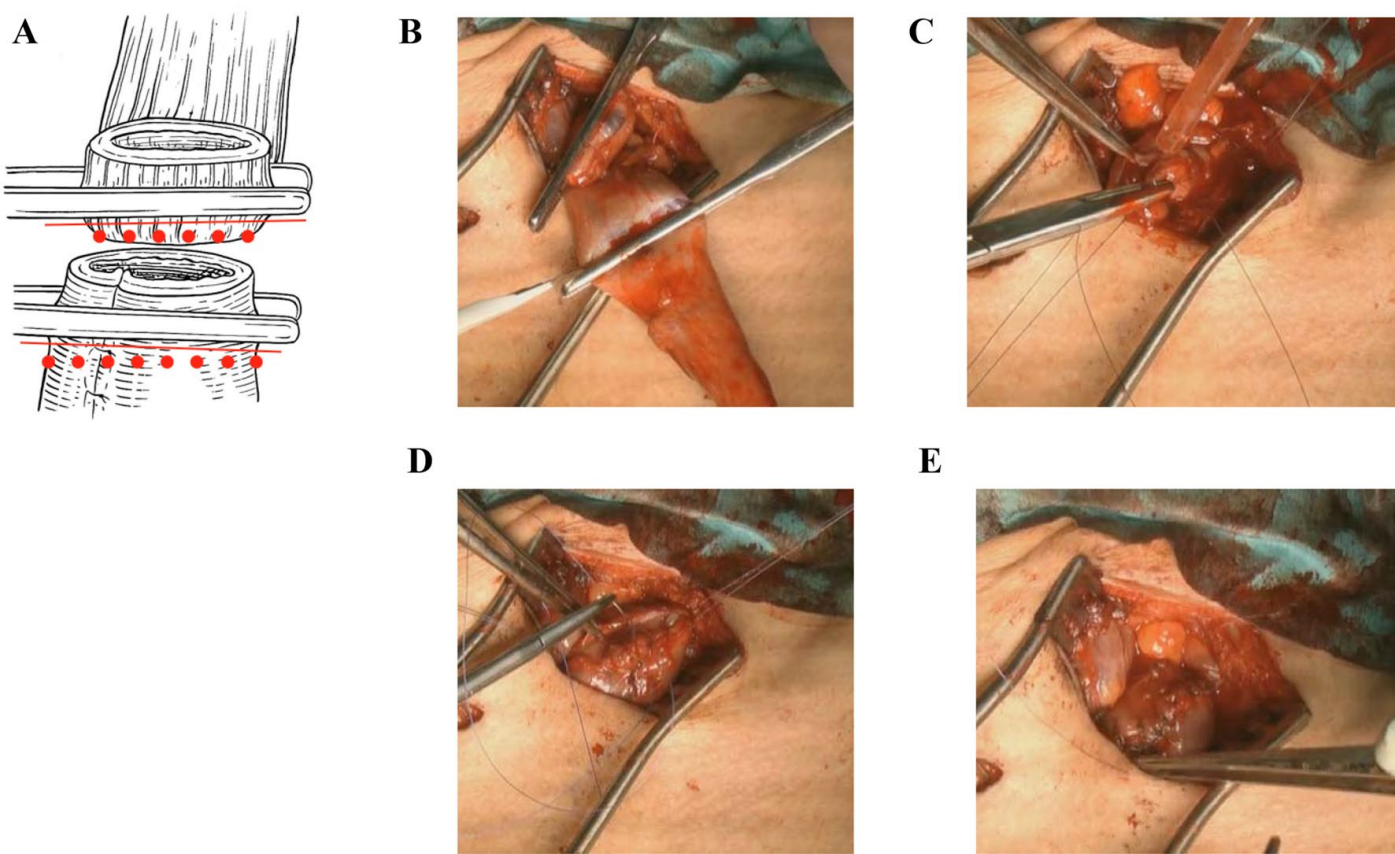
Our procedure for layer-to-layer handsewn anastomosis. Stumps of the esophagus and gastric conduit are grasped with straight grasping forceps. To avoid interrupting blood flow or crushing the tissues, anastomotic lines are set under the forceps (**A**). After cutting the posterior wall muscular layer with a scalpel (**B**), the posterior wall muscular layer is interrupted sutured using VICRYL Plus (ETHICON). The mucosal layer is then cut all the way around under the straight grasping forceps using scissors, and the inner cavity is opened. The posterior wall mucosal layer is running sutured to the anterior wall mucosal layer using PDSII or LACLON while monitoring blood flow in the esophagus and gastric conduit (**C**). Ligation of running suture is made in the submucosal layer to avoid placing the ligation in the inner cavity (**D**). Finally, the anterior wall muscular layer is interrupted sutured using VICRYL Plus (**E**).

Our surgical procedure for esophagectomy is reported elsewhere^9–11^. Briefly, our procedure for endo-to-endo, layer-to-layer handsewn anastomosis is as follows. Stumps of the esophagus and gastric conduit are grasped with straight grasping forceps. To avoid interrupting blood flow or crushing the tissues, anastomotic lines are set under the forceps (Fig. 4A). After cutting the posterior wall muscular layer with a scalpel (Fig. 4B), the muscular layer is interrupted sutured using VICRYL Plus (ETHICON). The mucosal layer under the straight grasping forceps is then cut all the way around using scissors, and the inner cavity is opened. The posterior wall mucosal layer is running sutured to the anterior wall mucosal layer using PDSII or LACLON while monitoring blood flow in the esophagus and gastric conduit (Fig. 4C). Ligation of the running suture is made in the submucosal layer to avoid placing the ligation in the inner cavity (Fig. 4D). Finally, the anterior wall muscular layer is interrupted sutured using VICRYL Plus (Fig. 4E). After washing the anastomosis with warm saline, the gastric conduit is pulled and straightened via an abdominal wound.

One of two surgeons with more than 15 years continuous experience as an esophageal surgeon at Akita University Hospital performed the anastomoses himself or carefully instructed four other surgeons, each of which had more than a total of 10 years experience as a visceral surgeon and as an esophageal surgeon. A PPI (Lansoprazole 30 mg/body) was administered intravenously before and after esophagectomy and then twice daily from postoperative day 1 to 8. Thereafter, the PPI (Lansoprazole 30 mg/body/day) was administered orally. Anastomotic leakage was assessed with esophagography using Gastrografin (BAYER, Osaka, Japan) on postoperative day 8. The preoperative, intraoperative and postoperative management was the same for both groups.

### pH monitoring

In the most recent four patients, pH at the anastomosis was monitored using a pH sensor (Digitrapper pH 400, Medtronic). The pH sensor was calibrated with pH 1 and pH 8 calibration solutions before insertion according to the manufacturer’s protocol. The sensor was attached to a nasogastric tube and inserted via a naris by the anesthesiologist soon after the handsewn anastomosis was completed. The estimated level of the anastomosis relative to the upper edge of the sternum was predictable during surgery, and chest X-rays confirmed the placement of channels 1 and 2 on the esophageal and gastric conduit sides after surgery. Monitoring began soon after the surgery and was continued for 24 h. Minimum pH, maximum pH, and the percentages of time pH was lower than 4 or higher than 8 on the esophageal side (channel 1) and gastric conduit side (channel 2) of the anastomosis were determined.

### Statistical analysis

All statistical analyses were performed using JMP12 (Version 12.2.0). Data are presented as the mean ± standard deviation. To evaluate differences between the PDSII and LACLON groups, the Mann–Whitney–Wilcoxon test (for continuous variables) and Fisher’s exact tests (for categorical variables) were used. Values of *P* < 0.05 were considered significant.
